# Supplementary material for: Polygenic contribution to the relationship of loneliness and social isolation with schizophrenia
Source: Nat Commun. 2022 Jan 10;13:51. doi: 10.1038/s41467-021-27598-6 (PMC8748758; doi:10.1038/s41467-021-27598-6)
Supplement: Supplementary file 1 — Supplementary Information [file 41467_2021_27598_MOESM1_ESM.pdf]

SUPPLEMENTARY INFORMATION

Polygenic contribution to the relationship of loneliness and social isolation with schizophrenia.

Table of Contents

Supplementary Methods 1. Participants and QC filtering 3

Supplementary Methods 2. Summary statistics from loneliness and isolation traits 4

Supplementary Methods 3. Polygenic score predictions 4

Supplementary Methods 4. Genomic dissection of schizophrenia based on LNL-ISO and stratified-polygenic score predictions 6

Supplementary Methods 5. LD-score regression (LDSC) and partitioning SNP heritability 7

Supplementary Methods 6. Partial genetic correlations between SCZ and related traits based on LNL-ISO annotations 9

Supplementary Methods 7. Bidirectional Causal Analyses: Two-sample Mendelian Randomization 10

Supplementary Figure 1. Polygenic score predictions of LNL-ISO ($PGS_{LNL-ISO}$) and its constituent phenotypes (see legend) on an independent schizophrenia case-control sample (N_{SCZ} = 1927; N_{HC} = 1561).. 13

Supplementary Figure 2. Partitioned heritability in annotations encompassing variants associated with LNL-ISO: comparison of enrichments across SCZ[CONC] and SCZ[DISC]. 14

Supplementary Figure 3. Results from the partitioned heritability analysis with LDSR for tissue and cell-type enrichment ………..…………………………………………………………… 15

Supplementary Figure 4. Density plot for sex comparison of PGS_{SCZ} predictions with alternative prevalence estimates ..……..…………………………………………………………… 16

Supplementary Figure 5. Results from Mendelian Randomization with IVW and WM methods and sensitivity tests of Loneliness and isolation traits and Schizophrenia liability ………………………………………………………………………………………………..…….. 17

Supplementary Figure 6. Single-SNP effect and leave-one-out sensitivity tests of Mendelian Randomization analyses of LNL-ISO and Loneliness UKBB on schizophrenia liability…………..…………………………………...............…………………………..… 19

Supplementary Methods 1. Participants and QC filtering

The CIBERSAM (Spain, https://www.cibersam.es/en) consortium collected blood samples from 2,145 subjects with a diagnosis of DSM-IV schizophrenia spectrum disorders or schizoaffective disorder (SCZ) and 1,711 healthy controls (HC) at 8 different hospitals across Spain. Individual genetic data was obtained by genotyping as part of the third wave of the schizophrenia genome wide association study (GWAS) performed by the Psychiatric Genomics Consortium (PGC).

After QC filtering, genetic data from 1,927 SCZ (65% males) and 1,561 HC (55% males) were used for the subsequent analyses. All remaining subjects were of European ancestry. Average age at inclusion was 33.15 years old.

Quality Control and imputation

We followed PGC-SZ2 criteria in applying QC filters[1]. Only autosomal genetic data was used. Briefly, consecutive filters were applied as follows:

- SNP missingness across samples < 0.05 (Before sample removal)

- Removal of subjects with a SNP missingness > 0.02.

- Autosomal heterozygosity deviation (|F het | < 0.2)

- SNP missingness < 0.02 (after sample removal)

- Difference in SNP missingness between SZ and HC < 0.02

- Hardy-Weinberg (HW) equilibrium ($P > 10^{-6}$ in HC or $P > 10^{-10}$ in SZ)

In order to gain genetic information beyond genotyped variants, we used the Michigan imputation server (https://imputationserver.sph.umich.edu/index.html). We retained a total of 9,340,561 variants with imputation quality > 0.3 and MAF > 0.01 and 3,489 individuals (1927 SZ and 1,561 HC).

Multidimensional scaling (MDS) was used to generate ancestry MDS covariates after imputation using variants with high imputation quality (INFO >0.8), MAF > 0.01 and after removal of major histocompatibility complex variation (from 26Mb to 33Mb of chromosome 6). SNP were pruned using PLINK v.1.9 with r2 < 0.1 in 500 SNP (*--indep-pairwise 500 1 0.1*). We used the 10 first MDS components as covariates for polygenic scores prediction in this case - control cohort.

Supplementary Methods 2. Summary statistics from loneliness and isolation traits

Summary statistics for the previously explored[2] loneliness and social isolation composite (LNL-ISO) were downloaded from the available repository (https://doi.org/10.17863/CAM.23511). The authors performed a combined multi-trait GWAS (MTAG)[3] in the UK Biobank study yielding an effective sample size of 487,647 individuals. Briefly, they combined summary genetic data from three GWAS at the UK Biobank sample, derived from three questions related to loneliness and social isolation:

(a) 'Do you often feel lonely?', as if individuals answered 'yes' (recorded as cases) or 'no' (controls)

(b) A composite variable based on the questions 'Including yourself, how many people are living together in your household?' and ''How often do you visit friends or family or have them visit you?' (cases were defined as those who lived alone and who indicated that they either never visited or had no friends or family outside their household; controls were defined as those who either did not live alone or had friends who visited at least once a week).

(c) A variable representing the quality of social interactions 'How often are you able to confide in someone close to you?' (cases were defined as those who answered 'Never or almost never', controls were defined as those who answered 'Almost daily').

Summary data from the latest UK biobank (UKBB) results regarding loneliness and isolation traits, from which LNL-ISO MTAG was originally created, were used: Loneliness (https://nealelab.github.io/UKBB_ldsc/h2_summary_2020.html), ability to confide (https://nealelab.github.io/UKBB_ldsc/h2_summary_2110.html) and social support measured by number of family and friend visits (https://nealelab.github.io/UKBB_ldsc/h2_summary_1031.html) and number of people living in household (https://nealelab.github.io/UKBB_ldsc/h2_summary_709.html).

Supplementary Methods 3. Polygenic score predictions

We filtered discovery datasets for polygenic score calculations for imputation quality score > 0.8, include only biallelic variation and exclude indels. Correlated SNPs due to linkage disequilibrium (LD) were removed using PLINK 1.9 clumping algorithm ($r^2 > 0.1$; window size = 500 kb). LD reference from the European subset of the 1000 Genomes reference panel was used from clumping. Due to the extremely complex LD pattern, genetic variants within Major Histocompatibility Complex (MHC) were removed (from 26Mb to 33Mb of chromosome

6). PLINK 1.9 was used to calculate PGS across schizophrenia patients and healthy controls weighted by the logOR in the discovery sample. Several P thresholds were used ($P<5 \times 10^{-8}$, 5×10^{-5}, 1×10^{-3}, 0.01, 0.05, 0.1, 0.2, 0.5 and 1).

Using LNL-ISO MTAG[2] and PGC-SZ2[1] GWAS summary statistics as discovery sample, and SCZ_CIBERSAM case-control cohort as target sample, we performed polygenic models (PGS_{SCZ} and $PGS_{LNL-ISO}$). PGS_{SCZ} were calculated using SNPs also present in LNL-ISO GWAS. Standardized PGS were calculated and significance was evaluated by logistic regression, using case-control status as dependent variable and sex, age and 10 first MDS ancestry components as covariates. We calculated explained variance attributable to PGS as the increase in Naggelkerke's pseudo-R^2 between a model with and without PGS variable. In PGS predictions with PGC-SZ2, Naggelkerke's pseudo-R^2 were converted to liability scale following the procedure proposed by Lee et al[4]. This way, we accounted for ascertainment bias due to oversampling of cases relative to the population prevalence and comparison with other studies can be done. A prevalence of schizophrenia in the general population of 1% was assumed for the calculation of the R^2 on the liability scale. All p-values were FDR corrected by Benjamini - Hochberg method (p_{FDR}). CI for the increase in R^2 was estimated through bootstrap resampling (N = 5000), applying the Normal Interval method, after checking the normality of the bootstrap distribution. PGC-SZ2[1] GWAS summary statistics have demonstrated a solid validity for being used as discovery samples in polygenic score predictions[5,6] Anyway, to compare predictions with another recent schizophrenia GWAS [7] including approximately 5000 new cases and 18000 controls to PGC2, PGS_{SCZ} were again calculated using SNPs from this later GWAS also present in LNL-ISO GWAS. No great improvements were observed in terms of explained variance.

In order to understand the direction of effect of the PGS across the different partitions, PGS_{SCZ} comparisons across ranked deciles were also performed. The target sample was first separated into 10 deciles of increasing PGS. The P-threshold with the lowest p-value was selected for each partition. The phenotype values of each decile were compared to those of the reference decile (the median decile (5th) was used as reference) one-by-one, with decile status as predictor of target phenotype (5th decile was coded 0 and tested decile 1) in a logistic regression model. OR values for each comparison were estimated from regression coefficients of these decile-status predictors. Sex, age and 10 first MDS ancestry components were used as covariates.

Supplementary Methods 4. **Genomic dissection of schizophrenia based on LNL-ISO and stratified-polygenic score predictions.**

In order to analyze the role of PGS_{SCZ} in the prediction of SCZ - HC status but taking into account the role of the genetic variation in LNL-ISO, we divided SCZ summary data into three different subsets of variants, according to their role in LNL-ISO. First, only variants included both in SCZ and LNL-ISO summary data were included (**SCZ[ALL]**; Final N_SNPs = 5,658,282). Second, predisposing variation to SCZ was divided into variants not associated with LNL-ISO (**SCZ[noLNL]**; $P_{LNL-ISO} > 0.05$; N_SNPs = 5,172,017) and variants associated with LNL-ISO (**SCZ[LNL]**; $P_{LNL-ISO} < 0.05$; N_SNPs = 486,265). This cutoff in $P_{LNL-ISO}$ was selected as P = 0.05 because predictions below this threshold reached the maximum value of explained variance, and $PGS_{LNL-ISO}$ predictions encompassing variants with $P_{threshold} > 0.05$ did not contribute to schizophrenia risk (R^2 (95% CI) = 0.052% (-0.09,0.10) at $P_{threshold} > 0.05$, $p = 0.57$); **supplementary data 1**). Third, SCZ[LNL] was again divided into those variants with concordant (**SCZ[CONC];** $P_{LNL-ISO} < 0.05$; $Beta_{SCZ} > 0$ & $Beta_{LNL-ISO} > 0$ / $Beta_{SCZ} < 0$ & $Beta_{LNL-ISO} < 0$; N_SNPs = 269,361) and discordant (**SCZ[DISC];** ; $Beta_{SCZ} > 0$ & $Beta_{LNL-ISO} < 0$ / $Beta_{SCZ} < 0$ & $Beta_{LNL-ISO} > 0$; N_SNPs = 216,904) sign of the effect allele between SCZ and LNL-ISO. In each dataset, we removed correlated SNPs due to linkage disequilibrium (LD) using the PLINK 1.9 clumping algorithm based on the selection of LD-independent SNP most associated with SCZ and we used independent variants within **SCZ[noLNL]** (N_clumpedSNPs = 169574), **SCZ[LNL]** (N_clumpedSNPs = 118,04), **SCZ[CONC]** (N_clumpedSNPs = 6468) and **SCZ[DISC]** (N_clumpedSNPs = 5,336) to calculate PGS on the SCZ_CIBERSAM case-control cohort (N_SCZ = 1,927; N_HC = 1,561). We calculated standardized PGS and evaluated significance by logistic regression, using case-control status as dependent variable and sex, age and 10 first MDS ancestry components as covariates. Explained variance attributable to PGS was calculated as the increase in R^2 between a model with and without PGS variable. Naggelkerke's pseudo-R^2 were converted to liability scale following the procedure proposed by Lee et al[4], assuming a prevalence of schizophrenia in the general population of 1%. All p-values were FDR corrected. CI for the increase in R^2 was estimated through bootstrap resampling (N = 5000), applying the Normal Interval method, after checking the normality of the bootstrap distribution.

Since loneliness perception and sociability have been reported to depend on sex (for instance, higher percentage of cases with loneliness autoperception in UKBB cohort are women (26.5% of women) instead of men (17.3% of men), we were motivated to analyze the sex differences in the impact of schizophrenia predisposing variation in the SCZ_CIBERSAM case-control cohort across the different subset of SNPs based on their role in LNL-ISO. So, we took a permutation-based approach: we performed bootstrap resampling (5,000 permutations) of 500 schizophrenia and 500 HC subjects across men and women separately within the

SCZ_CIBERSAM cohort, and calculated PGS and explained variance predictions by logistic regression and R^2 estimations on the liability scale. The P-threshold with the higher prediction in each sex was selected for each partition. No sex differences in various prevalence measures have been reported[8,9]. Therefore, we considered a prevalence of 1% for both sexes[10]. As a sensitivity analysis, we repeated the analyses to account for recent reports of different prevalence estimates in both sexes in the Spanish population[11] (prevalence in males = 0.0079 and females = 0.0045), with no significant changes in the results from the sex comparisons (**Supplementary Figure 3**). The process was repeated for each of the SNP subsets: SCZ[noLNL], SCZ[LNL], SCZ[CONC] and SCZ[DISC]. Variance explained in women and men was statistically compared with student t-tests.

Supplementary Methods 5. LD-score regression (LDSC) and partitioning SNP heritability

To obtain SNP heritability estimates related to annotations studied here, we followed a recommended procedure[12–14] (https://github.com/bulik/ldsc/wiki/Partitioned-Heritability).

First, we created per SNP annotation files (one per chromosome and desired annotation). Each file consisted of a row per SNP and a column for each sub-annotation (1 = a SNP is part of that sub-annotation). SNP that do not belong to the annotation were given a value of 0. Annotation files were created for:

- A) Each genome partition based on the relationship between SCZ and LNL-ISO, as done with polygenic score predictions: SCZ[noLNL],SCZ[CONC] and SCZ[DISC] (see **Supplementary Methods 3**).
- B) Adapted sub-annotation files for the intersection between the above described annotations (SCZ[noLNL] and SCZ[CONC]) and 10 whole tissue[13,14], 13 brain-related tissue (Brain GTEx from Finucane et al., 2018[14]) and 3 brain cell-type annotation files (Neuron, astrocytes and oligodendrocytes)[15] available at LDSC repository (http://data.broadinstitute.org/alkesgroup/LDSCORE/). SCZ[DISC] was not included here as the percentage of SNP-based heritability explained by this annotation was too low to assess enrichment within sub-annotations generated from it.

In order to generate annotation files from A, we generated bed files from the summary statistics overlap between SCZ and LNL-ISO as described before. We are aware of the risk of inflated heritability enrichment values of annotations encompassing variants significantly associated with LNL-ISO due to an increase in power. We made an additional analysis to rule out this possibility by comparing the enrichment of the same number of variants from SCZ[CONC] and SCZ[DISC]. Since SCZ[DISC] includes only 3.0% of SNPs in comparison to the 3.8%

of SCZ[CONC], the enrichment value of SCZ[DISC] was compared against the enrichments of 3.0% of SNPs from SCZ[CONC] partition, after 1000 permutations with replacement from the whole set of SNPs in SCZ[CONC]. The distribution of the enrichments of all the subsets of 3% of SNPs within SCZ[CONC] was compared to the real enrichment of SCZ[DISC]. The P value was calculated by dividing the number of permuted SCZ[CONC] sets that explained equal or lower variance than the true SCZ[DISC] set plus one by the total number of permutations plus one. The results of this comparison are shown in **Supplementary Figure 2**.

In the case of annotation files from B), bed files were initially generated by intersection between bed files from SCZ / LNL- ISO and bed-files from cell-type and tissue available annotations at LDSC repository. We performed bed file intersection with Bedtools[16] , using the *--intersectBed* command. Once we had bed files, we obtained annotation files using *--make-annot.py* and data files from phase 3 of the 1000 Genomes Project (1000 Genomes Project Consortium, 2015). We created a separated file for each annotation and chromosome.

We ran LDSC using associated data files from phase 3 of the 1000 Genomes Project[17]. LD scores were computed for each annotation file using the recommended parameters: 1-cM window (*--ld-wind-cm* 1), restriction to Hapmap3 SNPs and exclusion of major histocompatibility complex (MHC) region due to its high gene density and exceptional LD , as recommended by the developers[14]. The '*–overlap-annot*' argument and 1000 genomes phase 3 - based frequency files ('1000G_Phase3_frq' files via *--frqfile-chr* argument) and LD weights ('weights_hm3_no_hla' files via *--w-ld-chr* argument) were used for LD score calculations.

SCZ summary data from previous PGC GWAS[1] was used as input for heritability enrichment calculation. We used the *--munge-sumstats.py* command for formatting summary data, and only included SNPs present in HapMap 3. We also used another recent SCZ GWAS summary statistics[7] to check that no differences in the results were observed (**Supplementary table 3A**).

Partitioned LDSC computes the proportion of SNP heritability associated with each annotation column while taking into account all other annotations. This is performed by regression models using the estimated LD-scores jointly with other independent LD scores for baseline annotations to improve the model performance. We used the full baseline model v2.2, consistent of a full annotation column (1 per all SNPs) and 158 independent functional annotations, available at LDSC repository (https://data.broadinstitute.org/alkesgroup/LDSCORE/), as independent LD scores. Indeed, for the heritability estimation for annotations from B), based on intersection between SCZ/ LNL-ISO and tissue/cell-types, an additional independent LD-scores were used, as described next. As discussed with LDSC developers, the best way to obtain heritability estimates for a cell-type subannotation (for instance, neuronal cell-type annotation within SCZ[CONC]) is to include the annotation from which the cell-

type is sub-annotated (in the example case, the SCZ[CONC] annotations), apart from the independent annotations in the full baseline model.

Based on the proportion of total SNPs in an annotation and the percentage of the SNP heritability (h^2_{SNP}) in every case, LDSC calculates an enrichment score and an associated enrichment P value. For annotation files in B), we also estimated heritability for the intersection of the provided control files (these are annotations corresponding to all genes included in the study from which specifically expressed cell types are described) and the corresponding SCZ / LNL-ISO annotations. In the case of specific cell-types[15] and brain-expressed genes from GTEx, we also computed 'anti-target cell-type' enrichments. These are calculated as the intersection between the cell-type or brain specific annotation and the variation not belonging to the corresponding SCZ / LNL-ISO annotation. By doing this, we can compare specific cell-type enrichment within a particular SCZ / LNL - ISO annotation (for instance, neuronal enrichment within SCZ[CONC] annotation) to the cell-type enrichment out of this SCZ / LNL - ISO annotation (for the latter case, neuronal enrichment out of SCZ / LNL-ISO annotation). This also enables us to estimate the influence of LNL-ISO on a particular cell-type enrichment in SCZ.

To evaluate whether the cell-type enrichment within a particular LNL-ISO annotation is higher than the associated 'anti-target cell-type' enrichment, we applied LD score regression to specifically expressed genes (LDSC-SEG) using the *--h2-cts* argument to perform a one-sided t-test.

Supplementary Methods 6. Partial genetic correlations between SCZ and related traits based on LNL-ISO annotations.

To examine how the LNL-ISO based annotations (SCZ[noLNL], SCZ[CONC] and SCZ[DISC]) influence the correlation between schizophrenia and other related traits or disorder, we performed correlations but restricted ourselves to SNP subsets within each annotation. We selected a series of neuropsychiatric disorders and traits that have been previously demonstrated to be significantly correlated with SCZ and/or social isolation phenotypes. Available summary GWAS data from recent performed studies on the following disorders was obtained: major depression (MDD)[18], attention and deficit hyperactivity disorder (ADHD)[19], autism spectrum disorders (ASD)[20], Anxiety disorder (ANX)[21], Bipolar disorder (BIP)[22], obsessive compulsive disorder (OCD)[23], alcohol dependence disorder (ALC-DEP)[24] and cross-disorder (CROSS-DIS)[25] a meta-analysis conducted by a specific PGC workgroup, with a total sample of 232,964 cases and 494,162 controls, among eight mental disorders (anorexia nervosa, attention-deficit/hyperactivity disorder, autism spectrum disorder, bipolar disorder, major depression, obsessive-compulsive disorder, schizophrenia, and Tourette syndrome) to analyse

the pleiotropic contribution of different loci. We also studied related traits with available genetic data: neuroticism (NEUR)[26], depressive symptoms (DS)[27], subjective well-being (SWB)[27], psychotic experiences in the general population (PSY-EXP)[28], educational attainment (EA)[29] and body-mass index (BMI)[30].

Using GNOVA[29] , we studied partial correlations. This program implements an approach similar to LD score regression but is capable of working with SNP subsets, using LD-score generated files in LDSC for heritability estimation again in this step. GNOVA calculates the covariance between two disorders with a procedure that accounts for the LD structure and that is demonstrated to be robust to sample overlap (https://github.com/xtonyjiang/GNOVA). Full covariance between SCZ and the analyzed traits here independent of annotations was also performed with LD score regression program (by the *--rg* argument). Similar to LDSC procedure, the reference files were 1000 genomes phase 3 and hapmap3 files. We used the *--munge-sumstats.py* program from LDSC to generate the formatted summary statistics as input files for GNOVA. As recommended by the GNOVA developers, we used covariance instead of correlation estimates. Derived p-values were statistically corrected by Benjamini-Hochberg FDR procedure (p_{FDR}).

Supplementary Methods 7. Bidirectional Causal Analyses: Two-sample Mendelian Randomization

We used Mendelian Randomization to investigate the bidirectional causal relationships between Loneliness and Isolation traits and Schizophrenia liability.

Mendelian randomization (MR) is a statistical method for inferring causal effects that utilizes genetic variants as Instrumental Variables (IV) that are robustly associated with (a potentially modifiable) exposure to another outcome[31].

The selection of valid Instrumental variables in Two-sample Mendelian randomization relies on three main assumptions: 1) IV must be associated with the exposure (the relevance assumption); 2) IV must be independent of confounders and share no common mediator in the exposure-outcome relationship (the independence assumption); and 3) IV affects the outcome only through their effects on the exposure (exclusion restriction assumption)[32,33].

Guidelines recommend the IVW method with multiplicative random-effects as the primary analysis method for use with summarized data, because it is the most efficient analysis method with valid instrumental variables. However, results may be biased if not all assumptions are met, and some of the IVs are considered to be invalid. These assumptions are very restrictive, so different methods have been developed to deal with the violation of these assumptions, especially to control for horizontal pleiotropy. Several sensitivity analyses in which

heterogeneity and pleiotropy are measured could be used to test these assumptions[32]. Horizontal pleiotropy is a major threat to the validity of an MR analysis. We used different MR methods to deal with possible biases, including MR-PRESSO and a recently developed method (CAUSE) to account for correlated and uncorrelated pleiotropy[34].

We used the data of "Loneliness UKBB", "Frequency of family visits", "Number of people in household" and "able to confide" from MR-BASE[35] (https://gwas.mrcieu.ac.uk), based on an API wrapper (https://mrcieu.github.io/ieugwasr/) integrated in TwosampleMR package (https://mrcieu.github.io/TwoSampleMR)[27], to conduct MR isolation-traits analyses. The latest GWAS analysis of IEU GWAS consortium[36] were used and summary data have been obtained of GWAS VCF files with the gwasvcf R package (https://github.com/mrcieu/gwasvcf)

The five standard MR methods (Inverse Variance Weighted (IVW)[31], Weighted Median[37], MR-Eggers[38], Simple Mode and Weighted mode[39]) were conducted in the R package TwoSampleMR v.0.5.3 (https://github.com/mrcieu/TwoSampleMR) with the default settings[35].

TwoSampleMR used a default genome-wide significant SNP threshold at $p < 5 \times 10^{-8}$, LD-Clumping R2 threshold of 0.001 and a window of 10000kb. In the case of *number of household* we used a p<5 x 10 -6 due to the lack of a sufficient number of instrumental variables (IV) at the default threshold.

Presence of pleiotropy was examined by the MR–Egger intercept test, where a significant non-zero intercept ($p < 0.05$) indicates horizontal pleiotropy. We also implemented the heterogeneity tests (IVW and Egger Cochran 's Q statistic statistics) and other sensitivity analyses such as the leave-one-out test and Single SNP test[32] (See **Supplementary data 7** and **Supplementary figure 5-6**).

Additionally, we conducted Mendelian Randomization Pleiotropy RESidual Sum and Outlier (MR-PRESSO)[40] (https://github.com/rondolab/MR-PRESSO) for accounting for horizontal pleiotropy by outliers removal. Outliers are variants showing possible evidence of horizontal pleiotropy. MR-PRESSO uses RADIAL MR test to identify outliers and remove them. We implemented MR-PRESSO with a wrapped function of TwoSampleMR with NbDistribution = 1000 and DistortionTest Significative Threshold = 0.05 parameters. MR-PRESSO β-Effects shown in Table 1 were estimated after removing the outliers.

Finally, we used a MR latent-model method -CAUSE-[34] that includes all variables rather than only the most strongly associated with the exposure and takes into account both correlated and uncorrelated pleiotropy. CAUSE uses a mixture model for all the variants and the effect estimates and standard errors measured in GWAS

of the two traits. If M is the mediator(exposure) and Y (outcome), where γ(gamma) is the true causal effect, Z_j is an indicator that variant G_j is a correlated pleiotropic and θ_j is an indicator of uncorrelated pleiotropy, then:

$$\beta_{Y,j} = \gamma\beta_{M,j} + Z_j\eta\beta_{M,j} + \theta_j$$

Cause estimates posterior distributions of γ, η and q and compare the fit of posteriors from models with and without a causal effect. For this, CAUSE compares a model in which the causal effect is fixed at zero (the sharing model) to a model that allows a nonzero causal effect (the causal model). CAUSE uses the expected log pointwise posterior density (ΔELPD), a Bayesian model comparison approach to compare between the two models. We used the recommended default parameters of LD-pruning r2 = 0.1 and the Beta (1,10) prior distribution for q in our analyses (for more details of the method, see (https://jean997.github.io/cause/).

We applied multiple testing corrections by Benjamini-Hochberg FDR to all the results from the MR analyses (P_{FDR} < 0.05).

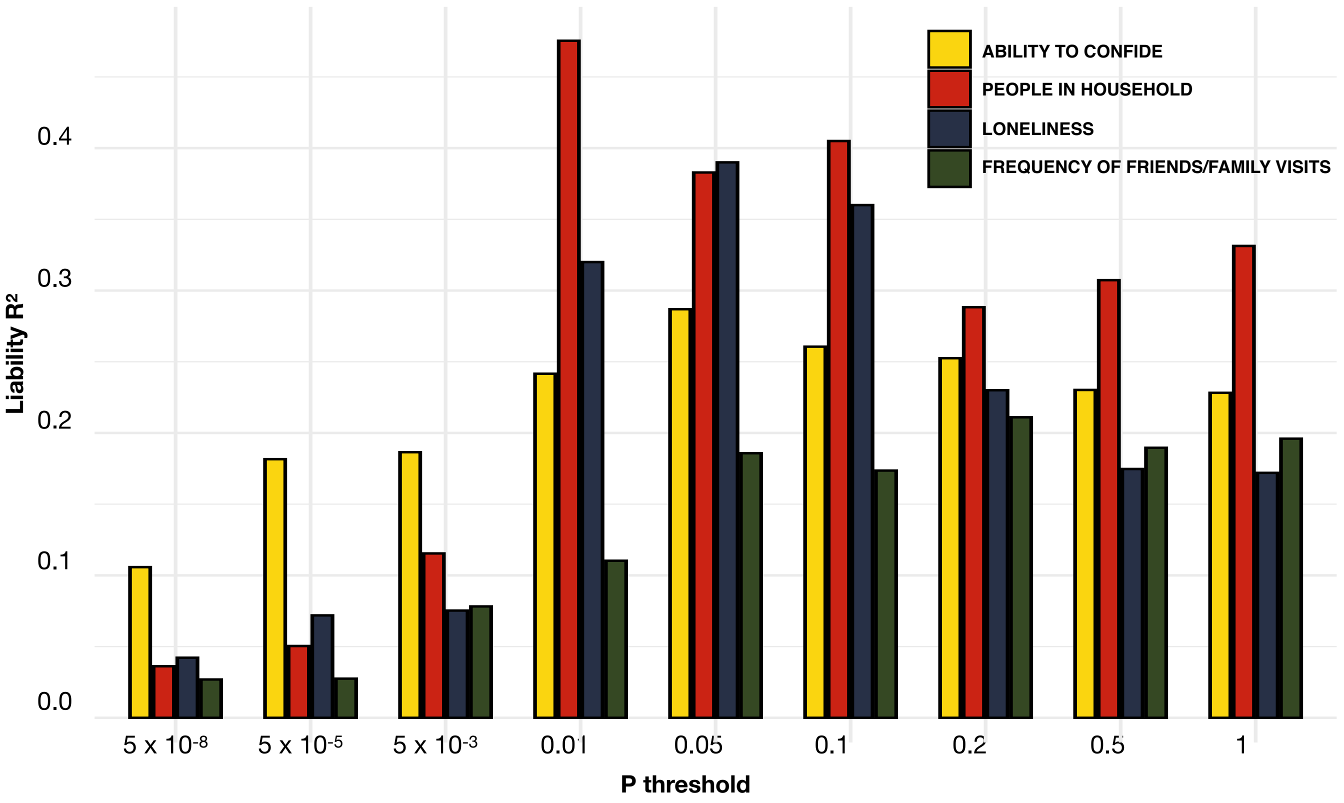

Supplementary Figure 1. Polygenic score predictions of LNL-ISO ($PGS_{LNL-ISO}$) and its constituent phenotypes (see legend) on an independent schizophrenia case-control sample (N_{SCZ} = 1927; N_{HC} = 1561). Explained variance attributable to PGS was calculated as the increase in Nagelkerke's pseudo-R2 between a linear model with and without the PGS variable. The R^2 proposed by Lee (Lee et al., 2012) was used to estimate the proportion of variance explained by the PGS on the liability scale corrected for ascertainment bias due to oversampling of cases relative to the population prevalence (Prevalence of LNL-ISO constituent phenotypes was estimated based on the case-control definition for each phenotype in the combined multi-trait GWAS (MTAG)[3] (see Supplementary Methods 2 for a full description)). Results were obtained from the binomial logistic regression of SCZ phenotype on PGS, accounting for LD and including sex and 10 MDS ancestry components as covariates.

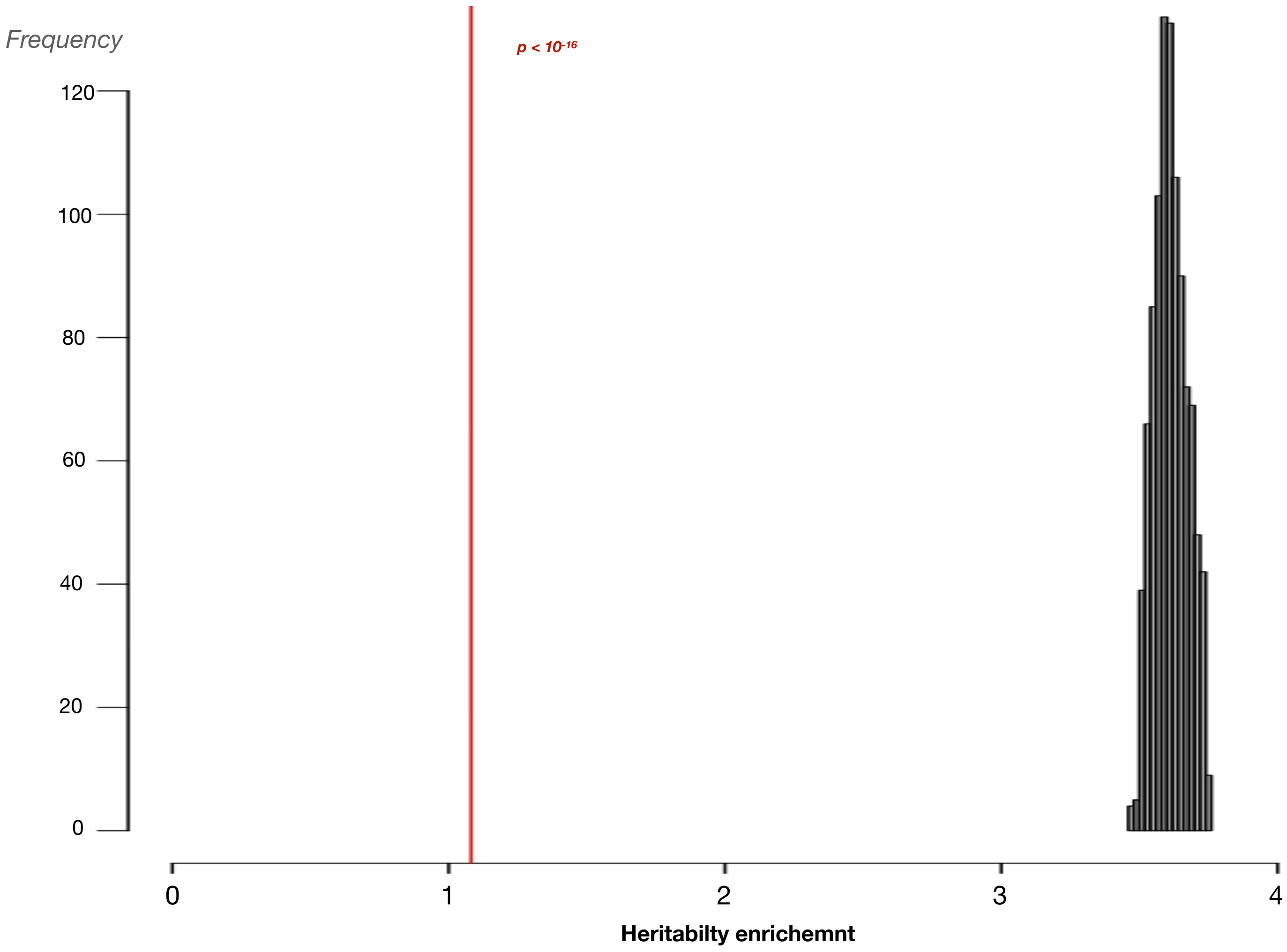

Supplementary Figure 2. Partitioned heritability in annotations encompassing variants associated with LNL-ISO: comparison of enrichments across SCZ[CONC] and SCZ[DISC]. Comparison of the heritability enrichment (LDSR) in schizophrenia of the same number of variants from SCZ[CONC] and SCZ[DISC]. To compare the similar number of SNPs from SCZ[CONC] and SCZ[DISC], the enrichment value of SCZ[DISC] (in red) was compared against the 1000 enrichments of 3.0% of SNPs from SCZ[CONC] partition, after 1000 permutations with replacement from the whole 3.8% of SCZ[CONC] SNPs (histogram). The distribution of enrichments of 3% of SNPs within SCZ[CONC] was statistically compared to the real enrichment of SCZ[DISC] with one-sided t-tests. Derived p-value is displayed ($p < 10^{-16}$).

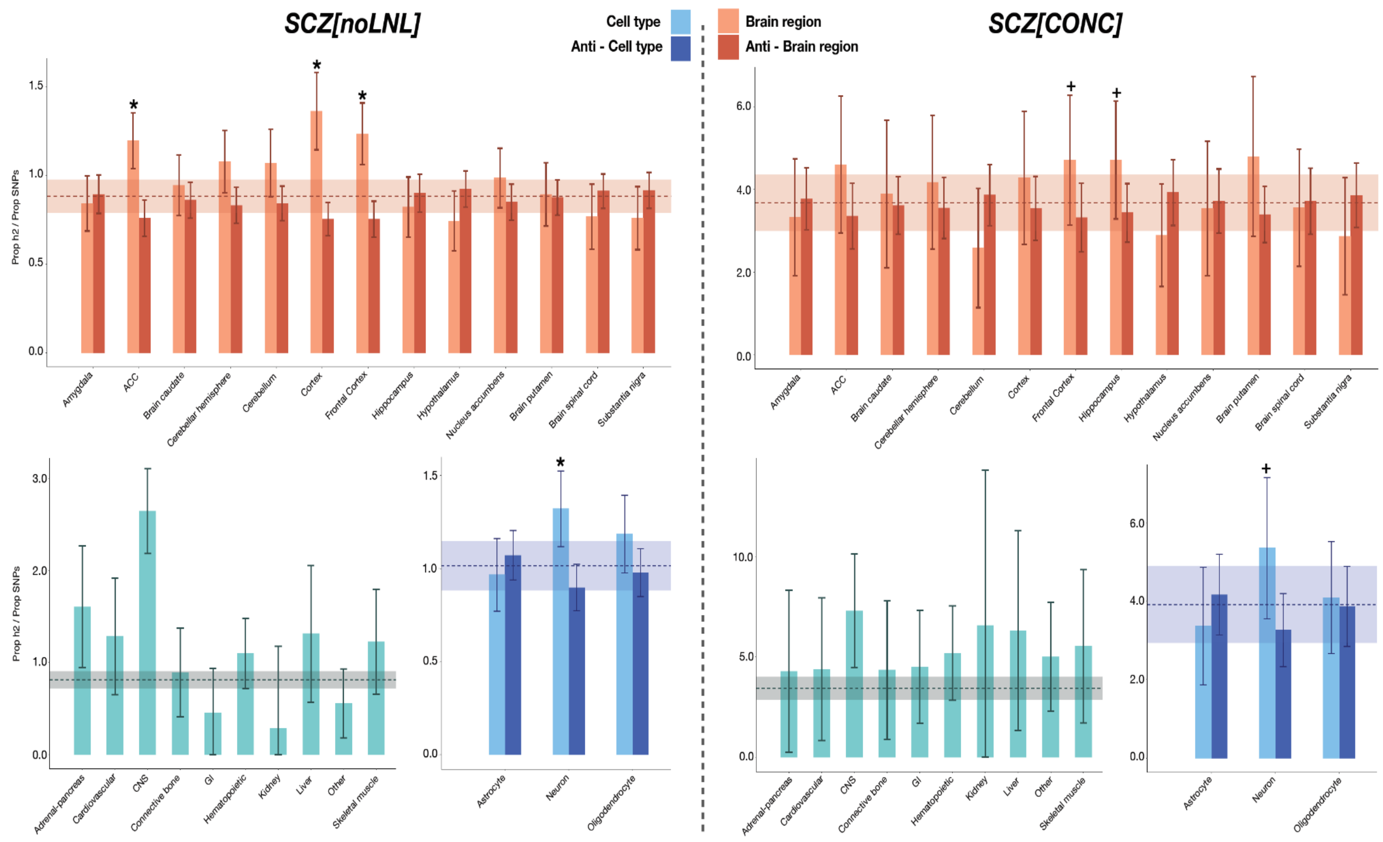

Supplementary Figure 3. Results from the partitioned heritability analysis with LDSR for tissue and cell-type enrichment.

Green, red and blue bars represent heritability enrichment for 10 tissues (Bullik-Sullivan et al., 2015; http://data.broadinstitute.org/alkesgr oup/LDSCORE/), 13 brain tissues from GTEx (Bullik-Sullivan et al., 2015; http://data.broadinstitute.org/alkesgroup/LDSCORE/) and 3 brain cell-types from Cahoy et al. (2008) (Finucane et al., 2018; http://data.broadinstitute.org/alkesgroup/LDSCORE/) across i) SCZ[noLNL] (i.e., SNPs from SCZ GWAS not associated with LNL-ISO (P > 0.05)) and ii) SCZ[CONC] (i.e., SNPs from SCZ GWAS associated with LNL-ISO (P < 0.05)) genome annotations. In brain tissue and cell type graphs, target tissue is compared to 'anti-target tissue' enrichment (see Supplementary Methods). Proportion of SNP-based heritability (h^2_{SNP}) and heritability enrichment (h^2_{SNP}/N_{SNP}) of the annotations were estimated by LD-score regression (LDSR) software. Error bars displayed represent 95% confidence intervals using standard errors (estimation +/- 1.96*SE). One-sided t-tests for evaluating whether the cell-type enrichment within a particular LNL-ISO annotation is higher than the associated 'anti-target cell type' (cell-type or brain tissue) or 'control tissue' (10 tissues) were also performed. * Significant results in the comparison between target and anti-target/control tissue enrichment after Benjamini-Hochberg FDR correction ($p_{FDR} < 0.05$). + Significant results at the non-corrected level in the target tissue vs anti- target/control tissue enrichment comparison ($p < 0.05$). Dashed lines and shading around them represent the enrichment and its standard deviation for the control tissue in each case. ACC: Anterior cingulate cortex; CNS: Central nervous system.

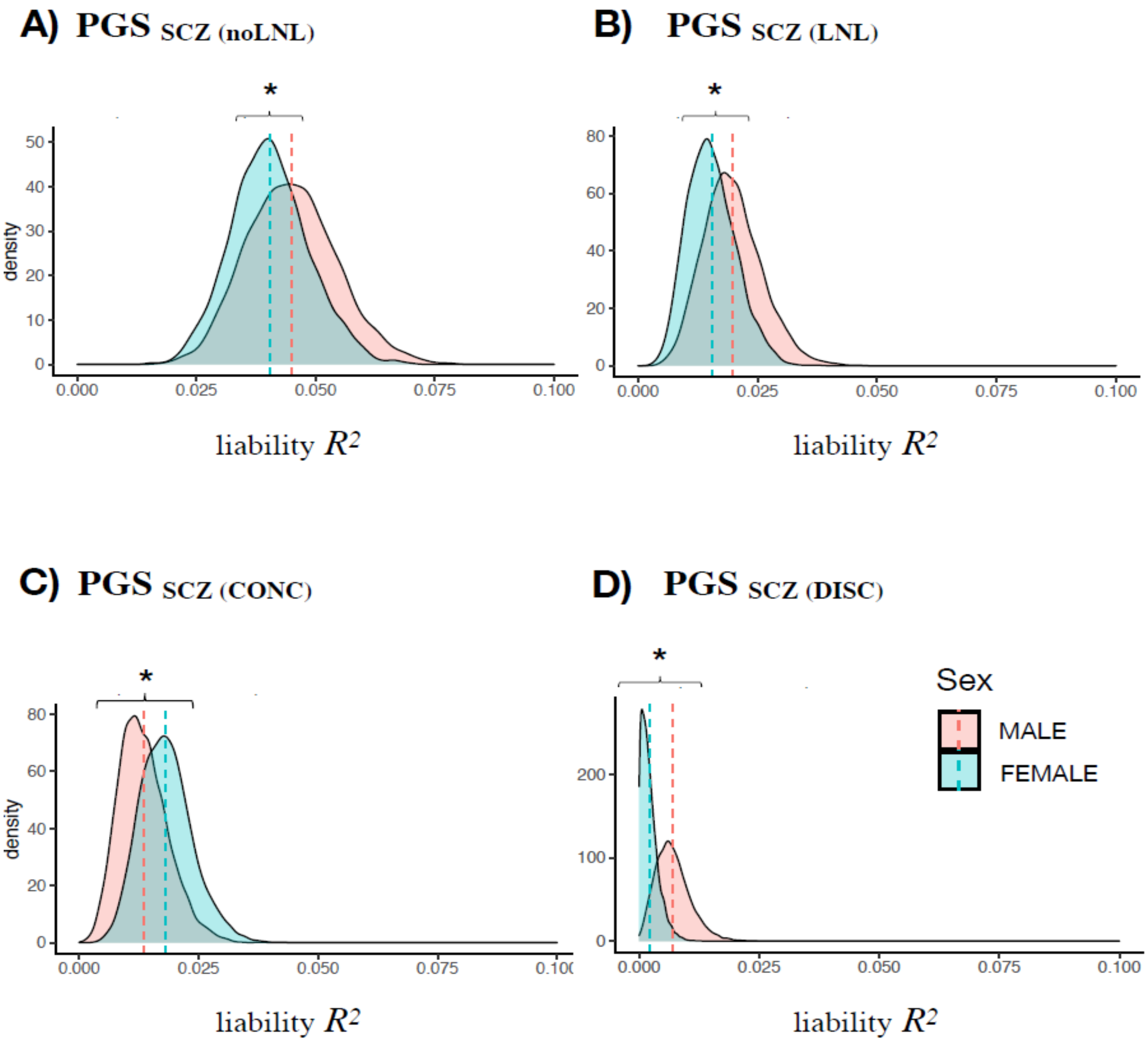

Supplementary Figure 4. Density plot for sex comparison of PGS_{SCZ} predictions with alternative prevalence estimates. PGS_{SCZ} predictions in case - control subsamples after bootstrap resampling (5,000 permutations) of 500 schizophrenia patients (SCZ) and 500 healthy controls (HC) (selected from the overall CIBERSAM case-control sample) were performed in males (NSCZ = 1253; NHC= 859) and females (NSCZ=674; NHC= 702), separately. Mean SCZ-HC variance explained by PG_{SSCZ} in the liability scale (prevalence of 0.0047 for females and 0.0079 for males[11] in males and females was compared for predisposing variation within A) SCZ[noLNL] , B) SCZ[LNL], C) SCZ[CONC], and D) SCZ[DISC]. Variance explained in females and males was statistically compared with two-sided t-tests and marked with an asterisk when it is significantly different in men and women ($p < 0.05$).

A

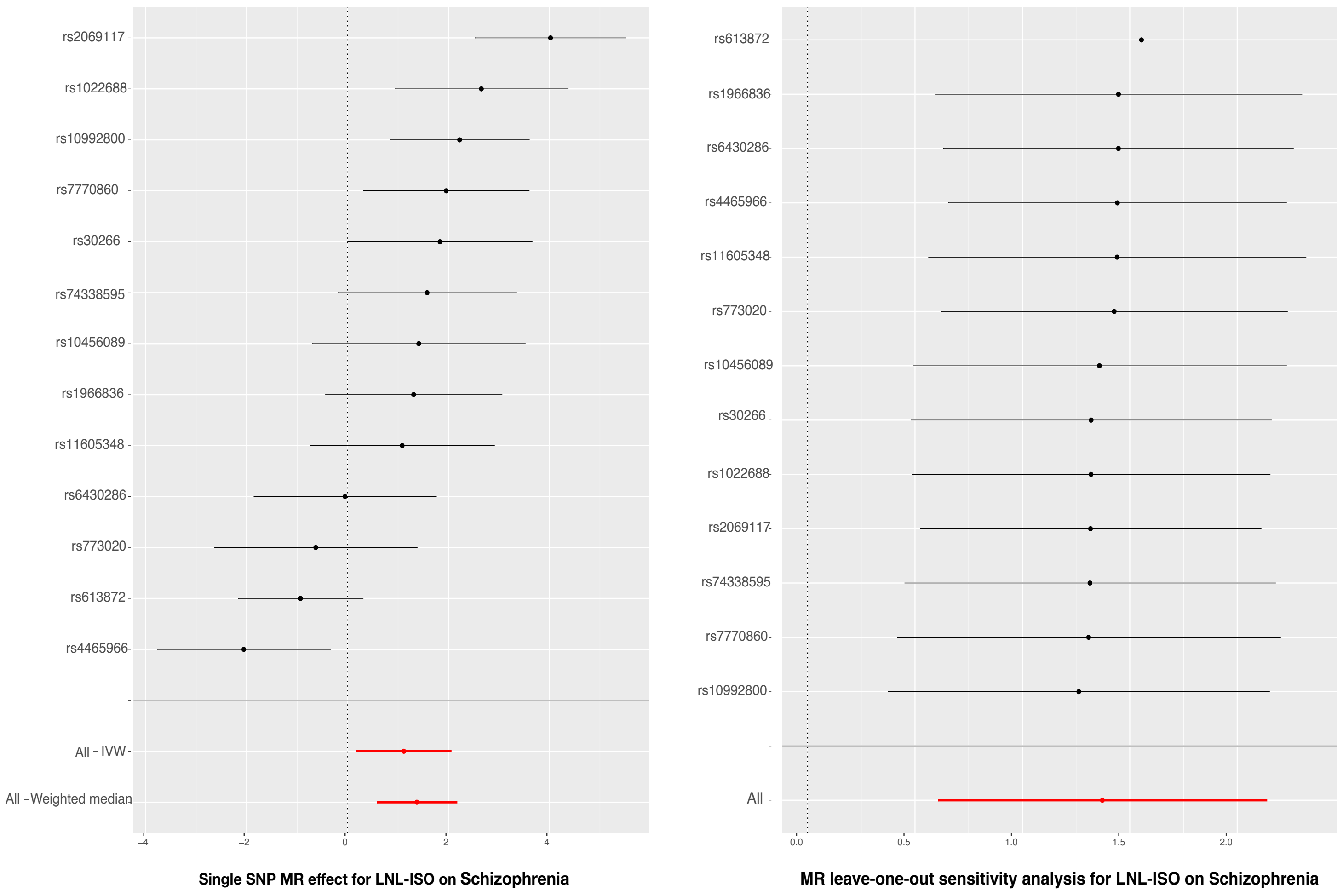
rs2069117
rs1022688
rs10992800
rs7770860
rs30266
rs74338595
rs10456089
rs1966836
rs11605348
rs6430286
rs773020
rs613872
rs4465966
All - IVW
All - Weighted median
-4
-2
0
2
4
Single SNP MR effect for LNL-ISO on Schizophrenia
rs613872
rs1966836
rs6430286
rs4465966
rs11605348
rs773020
rs10456089
rs30266
rs1022688
rs2069117
rs74338595
rs7770860
rs10992800
All
0.0
0.5
1.0
1.5
2.0
MR leave-one-out sensitivity analysis for LNL-ISO on Schizophrenia

B

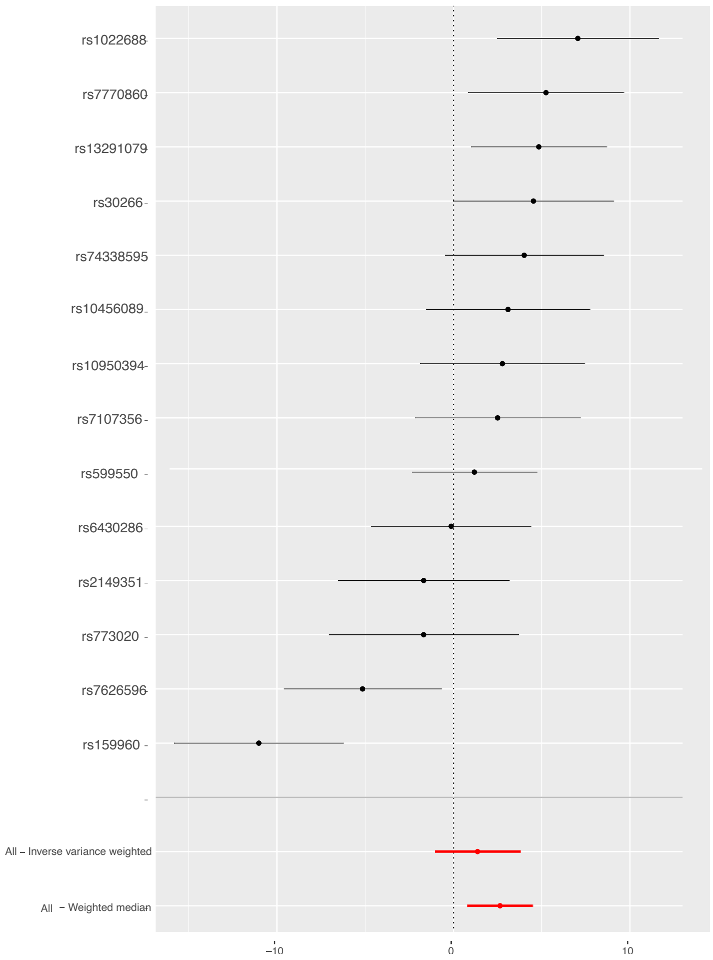

Single SNP MR effect for Loneliness UKBB on Schizophrenia

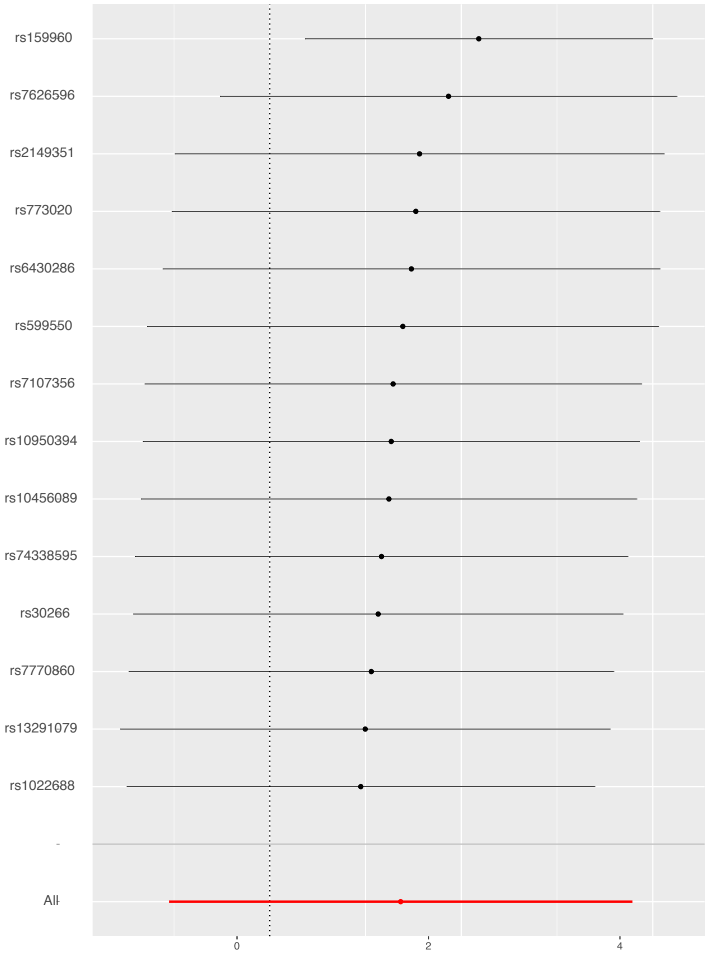

MR leave-one-out sensitivity analysis for Loneliness UBB on Schizophrenia

Supplementary Figure 5. Single-SNP effect and Leave-one-out Sensitivity test of Mendelian Randomization analyses of LNL-ISO and Loneliness UKBB on Schizophrenia liability. A) Forest Plots of Single SNP effects(left) and Leave-one-out analysis (right) of LNL-ISO against Schizophrenia liability. **B)** Forest Plots of Single SNP effects(left) and Leave-one-out analysis (right) of Loneliness UKBB against Schizophrenia liability. Note that a single variable (rs159960) in Loneliness UKBB causes a greater discordant effect on schizophrenia than the rest of the instruments in the leave-one-out test. In this scenario, MRPRESSO produced a significant result after removing outliers (See Table 1).

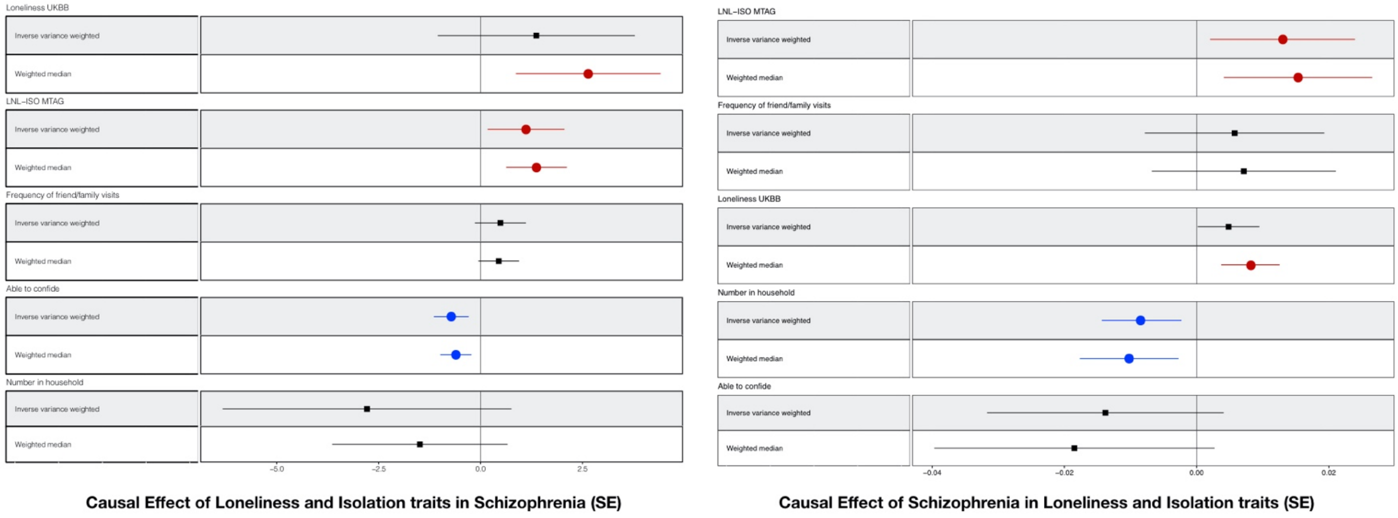

Supplementary Figure 6. Results from Bidirectional Mendelian Randomization with IVW and WM methods of loneliness and isolation traits and schizophrenia liability. One-to-many forest plots of the results of bidirectional causality analysis with two MR methods. Red dots represent a significant positive causal association. Blue dots represent a negative or protective causal association.

Supplementary references

1. Schizophrenia Working Group of the Psychiatric Genomics Consortium. Biological insights from 108 schizophrenia-associated genetic loci. *Nature* **511**, 421–427 (2014).
2. Day, F. R., Ong, K. K. & Perry, J. R. B. Elucidating the genetic basis of social interaction and isolation. *Nat Commun* **9**, 2457 (2018).
3. Turley, P. *et al.* Multi-trait analysis of genome-wide association summary statistics using MTAG. *Nat. Genet.* **50**, 229–237 (2018).
4. Lee, S. H., Goddard, M. E., Wray, N. R. & Visscher, P. M. A better coefficient of determination for genetic profile analysis. *Genet Epidemiol* **36**, 214–224 (2012).
5. Guloksuz, S. *et al.* Examining the independent and joint effects of molecular genetic liability and environmental exposures in schizophrenia: results from the EUGEI study. *World Psychiatry* **18**, 173–182 (2019).
6. Davies, R. W. *et al.* Using common genetic variation to examine phenotypic expression and risk prediction in 22q11.2 deletion syndrome. *Nat Med* **26**, 1912–1918 (2020).
7. Pardiñas, A. F. *et al.* Common schizophrenia alleles are enriched in mutation-intolerant genes and in regions under strong background selection. *Nat Genet* **50**, 381–389 (2018).
8. Charlson, F. J. *et al.* Global Epidemiology and Burden of Schizophrenia: Findings From the Global Burden of Disease Study 2016. *Schizophr Bull* **44**, 1195–1203 (2018).
9. Perälä, J. *et al.* Lifetime prevalence of psychotic and bipolar I disorders in a general population. *Arch Gen Psychiatry* **64**, 19–28 (2007).
10. McGrath, J., Saha, S., Chant, D. & Welham, J. Schizophrenia: a concise overview of incidence, prevalence, and mortality. *Epidemiol Rev* **30**, 67–76 (2008).
11. Orrico-Sánchez, A., López-Lacort, M., Muñoz-Quiles, C., Sanfélix-Gimeno, G. & Díez-Domingo, J. Epidemiology of schizophrenia and its management over 8-years period using real-world data in Spain. *BMC*

Psychiatry **20**, 149 (2020).

12. Bulik-Sullivan, B. K. *et al.* LD Score regression distinguishes confounding from polygenicity in genome-wide association studies. *Nature Genetics* **47**, 291–295 (2015).
13. Finucane, H. K. *et al.* Partitioning heritability by functional annotation using genome-wide association summary statistics. *Nat. Genet.* **47**, 1228–1235 (2015).
14. Finucane, H. K. *et al.* Heritability enrichment of specifically expressed genes identifies disease-relevant tissues and cell types. *Nat Genet* **50**, 621–629 (2018).
15. Cahoy, J. D. *et al.* A Transcriptome Database for Astrocytes, Neurons, and Oligodendrocytes: A New Resource for Understanding Brain Development and Function. *J. Neurosci.* **28**, 264–278 (2008).
16. Quinlan, A. R. & Hall, I. M. BEDTools: a flexible suite of utilities for comparing genomic features. *Bioinformatics* **26**, 841–842 (2010).
17. Auton, A. *et al.* A global reference for human genetic variation. *Nature* **526**, 68–74 (2015).
18. Howard, D. M. *et al.* Genome-wide meta-analysis of depression identifies 102 independent variants and highlights the importance of the prefrontal brain regions. *Nat Neurosci* **22**, 343–352 (2019).
19. Demontis, D. *et al.* Discovery of the first genome-wide significant risk loci for attention deficit/hyperactivity disorder. *Nature Genetics* **51**, 63–75 (2019).
20. Grove, J. *et al.* Identification of common genetic risk variants for autism spectrum disorder. *Nat Genet* **51**, 431–444 (2019).
21. Otowa, T. *et al.* Meta-analysis of genome-wide association studies of anxiety disorders. *Mol Psychiatry* **21**, 1391–1399 (2016).
22. Stahl, E. A. *et al.* Genome-wide association study identifies 30 loci associated with bipolar disorder. *Nature Genetics* **51**, 793–803 (2019).
23. Arnold, P. D. *et al.* Revealing the complex genetic architecture of obsessive–compulsive disorder using meta-analysis. *Molecular Psychiatry* **23**, 1181–1188 (2018).

24. Walters, R. K. *et al.* Transancestral GWAS of alcohol dependence reveals common genetic underpinnings with psychiatric disorders. *Nature Neuroscience* **21**, 1656–1669 (2018).

25. Cross-Disorder Group of the Psychiatric Genomics Consortium. Electronic address: plee0@mgh.harvard.edu & Cross-Disorder Group of the Psychiatric Genomics Consortium. Genomic Relationships, Novel Loci, and Pleiotropic Mechanisms across Eight Psychiatric Disorders. *Cell* **179**, 1469-1482.e11 (2019).

26. Nagel, M. *et al.* Meta-analysis of genome-wide association studies for neuroticism in 449,484 individuals identifies novel genetic loci and pathways. *Nature Genetics* **50**, 920–927 (2018).

27. Okbay, A. *et al.* Genetic variants associated with subjective well-being, depressive symptoms, and neuroticism identified through genome-wide analyses. *Nature Genetics* **48**, 624–633 (2016).

28. Legge, S. E. *et al.* Association of Genetic Liability to Psychotic Experiences With Neuropsychotic Disorders and Traits. *JAMA Psychiatry* **76**, 1256–1265 (2019).

29. Lee, J. J. *et al.* Gene discovery and polygenic prediction from a genome-wide association study of educational attainment in 1.1 million individuals. *Nature Genetics* **50**, 1112–1121 (2018).

30. Yengo, L. *et al.* Meta-analysis of genome-wide association studies for height and body mass index in ∼700000 individuals of European ancestry. *Human Molecular Genetics* **27**, 3641–3649 (2018).

31. Davey Smith, G. & Hemani, G. Mendelian randomization: genetic anchors for causal inference in epidemiological studies. *Hum. Mol. Genet.* **23**, R89-98 (2014).

32. Burgess, S. *et al.* Guidelines for performing Mendelian randomization investigations. *Wellcome Open Res* **4**, 186 (2019).

33. Davies, N. M., Holmes, M. V. & Davey Smith, G. Reading Mendelian randomisation studies: a guide, glossary, and checklist for clinicians. *BMJ* **362**, k601 (2018).

34. Morrison, J., Knoblauch, N., Marcus, J. H., Stephens, M. & He, X. Mendelian randomization accounting for correlated and uncorrelated pleiotropic effects using genome-wide summary statistics. *Nat. Genet.* **52**, 740–747 (2020).

35. Hemani, G. *et al.* The MR-Base platform supports systematic causal inference across the human phenome. *eLife* **7**, e34408 (2018).

36. Ruth Mitchell, E. MRC IEU UK Biobank GWAS pipeline version 2. *data.bris* https://data.bris.ac.uk/data/dataset/pnoat8cxo0u52p6ynfaekeigi (2019) doi:10.5523/bris.pnoat8cxo0u52p6ynfaekeigi.

37. Bowden, J., Davey Smith, G., Haycock, P. C. & Burgess, S. Consistent Estimation in Mendelian Randomization with Some Invalid Instruments Using a Weighted Median Estimator. *Genet. Epidemiol.* **40**, 304–314 (2016).

38. Bowden, J., Davey Smith, G. & Burgess, S. Mendelian randomization with invalid instruments: effect estimation and bias detection through Egger regression. *Int J Epidemiol* **44**, 512–525 (2015).

39. Hartwig, F. P., Davey Smith, G. & Bowden, J. Robust inference in summary data Mendelian randomization via the zero modal pleiotropy assumption. *Int J Epidemiol* **46**, 1985–1998 (2017).

40. Verbanck, M., Chen, C.-Y., Neale, B. & Do, R. Detection of widespread horizontal pleiotropy in causal relationships inferred from Mendelian randomization between complex traits and diseases. *Nat. Genet.* **50**, 693–698 (2018).
