# Supplementary material for: Polygenic contribution to the relationship of loneliness and social isolation with schizophrenia
Source: Nat Commun. 2022 Jan 10;13:51. doi: 10.1038/s41467-021-27598-6 (PMC8748758; doi:10.1038/s41467-021-27598-6)
Supplement: Supplementary file 3 — Description of Additional Supplementary Files [file 41467_2021_27598_MOESM3_ESM.pdf]

Description of Additional Supplementary Files

File Name: Supplementary Data 1
Description: PGS predictions for **A)** Loneliness and social isolation (Day et al., 2018) and **B)** Loneliness (UKBB), Ability to confide, People in household and Frequency of family/friends visits phenotypes (UK Biobank) in the SCZ-HC cohort of the present study (N_{SCZ} =1927; N_{HC} = 1561). Explained variance attributable to PGS was calculated as the increase in Nagelkerke's pseudo-R^2 between a model with and without the PGS variable. Number of SNPs used in any case, after clumped, is also displayed (N_{SNP}). CI for the increase in R^2 was estimated through bootstrap resampling (N = 5000). P-value and its corrected value after Benjamini-Hochber FDR is also described. For the most significant value, 10000 permutations for the same number of variants were performed. P-value from permutation analysis is also displayed. Values in red are those significant in any case**.** **C)** PGS predictions were also performed for Loneliness and social isolation (Day et al., 2018) using only variants with P > 0.05.

File Name: Supplementary Data 2
Description: **A)** PGS_{SCZ} predictions in the SCZ-HC cohort of the present study (N_{SCZ} = 1927; N_{HC} = 1561) using different sets of SNPs based on their association with LNL-ISO (Day et al., 2018). Explained variance attributable to PGS was calculated as the increase in Nagelkerke's pseudo-R^2 between a linear model with and without the PGS variable. P-values were obtained from the binomial logistic regression of SCZ phenotype on PGS, accounting for LD and including sex, age and 10 MDS ancestry components. Then, the R^2 proposed by Lee (Lee et al., 2012) was used to estimate the proportion of variance explained by the PGS on the liability scale corrected for ascertainment bias due to oversampling of cases relative to the population prevalence (Prevalence SCZ = 0.01). Number of SNPs used in any case, after clumped, is also displayed (N_{SNP}). CI for the increase in R^2 was estimated through bootstrap resampling (N = 5000). SNP sets used are the followings:**A1)** SCZ[ALL]: All SNPs from SCZ GWAS (Ripke et al., 2014) also present in LNL-ISO GWAS (Day et al., 2018); **A2)** SCZ[noLNL]: SNPs from SCZ GWAS not associated with LNL-ISO (P > 0.05); **A3)** SCZ[LNL]: SNPs from SCZ GWAS associated with LNL-ISO (P < 0.05); **A4)** SCZ[CONC]: SNPs from SCZ GWAS associated with LNL-ISO (P < 0.05) and with allele concordance in direction of effects (Beta) in both GWAS; **A5)** SCZ[DISC]: SNPs from SCZ GWAS associated with LNL-ISO (P < 0.05) and with allele discordance in direction of effects (Beta) in both GWAS. **A6)** To compare predictions with another recent SCZ GWAS (Pardiñas et al., 2018), PGS were again calculated using SNPs from this SCZ GWAS also present in LNL-ISO GWAS (Day et al., 2018) (SCZ (ALL - CLOZUK)). R_2-liabilitues are expressed in percentage (%). **B)** Quantile plot of PGS_{SCZ} predictions from partitions described in Supplementary Data 2A. The target sample is separated into deciles of increasing PGS. The case-control status of each decile is compared to the median (5th) decile, one-by-one, using a logistic regression model with covariates. 95% CI and P-values were extracted from logistic predictions. Red P-values denote significant comparison after FDR-correction.

File Name: Supplementary Data 3
Description: **A)** Results from the partitioned heritability analysis with LDSR for the genome partitions based on the relationship between SCZ (Ripke et al., 2014 **(A1)**; Pardiñas et al., 2018 **(A2)**) and LNL-ISO (Day et al., 2018): SCZ[noLNL]: SNPs from SCZ GWAS not associated with LNL-ISO (P > 0.05); SCZ[CONC]: SNPs from SCZ GWAS associated with LNL-ISO (P <

0.05) and with allele concordance in direction of effects (Beta) in both GWAS; SCZ[DISC]: SNPs from SCZ GWAS associated with LNL-ISO (P < 0.05) and with allele discordance in direction of effects (Beta) in both GWAS. Prop_h2 is the amount of genetic heritability based on SNPs explained by each annotation. the enrichment and its the standard error are shown in each case. Proportion of SNP-based heritability (Prop_h2) and heritability enrichment of the three LNL-ISO based annotations (SCZ[noLNL] , SCZ[CONC] and SCZ[DISC]) in schizophrenia estimated by LD-score regression (LDSR) software. Enrichment – P-values and standard errors were calculated with block jackknife procedure. **B)** Results from the partitioned heritability analysis with LDSR for 10 tissues (Bullik-Sullivan et al., 2015; http://data.broadinstitute.org/alkesgroup/LDSCORE/) across SCZ[noLNL] (SNPs from SCZ GWAS not associated with LNL-ISO (P > 0.05)) and SCZ[CONC] (SNPs from SCZ GWAS associated with LNL-ISO (P < 0.05) and with allele concordance in direction of effects (Beta) in both SCZ and LNL-ISO). Prop_h2 is the amount of genetic heritability based on SNPs explained by each annotation. The enrichment and its standard error are shown in each case. Control annotations from the original study (Bullik-Sullivan et al., 2015) were also included. One-sided t-test for evaluating whether the cell-type enrichment within a particular LNL-ISO annotation is higher than the CONTROL tissue were performed (see supplementary methods). Tissues in bold red are those significant with higher enrichment than corresponding control tissue. Proportion of SNP-based heritability (Prop_h2) and heritability enrichment were estimated by LD-score regression (LDSR) software. **C)** Results from the partitioned heritability analysis with LDSR for 13 Brain tissues from GTEx (Bullik-Sullivan et al., 2015; http://data.broadinstitute.org/alkesgroup/LDSCORE/) across SCZ[noLNL] (SNPs from SCZ GWAS not associated with LNL-ISO (P > 0.05)) and SCZ[CONC] (SNPs from SCZ GWAS associated with LNL-ISO (P < 0.05) and with allele concordance in direction of effects (Beta) in both SCZ and LNL-ISO). Prop_h2 is the amount of genetic heritability based on SNPs explained by each annotation. Proportion of SNP-based heritability (Prop_h2) and heritability enrichment were estimated by LD-score regression (LDSR) software. One-sided t-test for evaluating whether the cell-type enrichment within a particular LNL-ISO annotation is higher than the associated 'anti-target tissue' were also performed (see supplementary methods). Control annotations from the original study (Bullik-Sullivan et al., 2015) were also included. Tissues in bold black are those with higher enrichment than corresponding anti-target tissue at nominal significance. Tissues in bold red are those with higher enrichment than corresponding anti-target tissue at significance after FDR correction. **D)** Results from the partitioned heritability analysis with LDSR for 3 Brain cell types from Cahoy et al. (2008) (Finucane et al., 2018; http://data.broadinstitute.org/alkesgroup/LDSCORE/) across SCZ[noLNL] (SNPs from SCZ GWAS not associated with LNL-ISO (P > 0.05)) and SCZ[CONC] (SNPs from SCZ GWAS associated with LNL-ISO (P < 0.05) and with allele concordance in direction of effects (Beta) in both SCZ and LNL-ISO). Prop_h2 is the amount of genetic heritability based on SNPs explained by each annotation. Proportion of SNP-based heritability (Prop_h2) and heritability enrichment were estimated by LD-score regression (LDSR) software. One-sided t-test for evaluating whether the cell-type enrichment within a particular LNL-ISO annotation is higher than the associated 'anti-target cell type' were also performed (see supplementary methods). Control annotations from the original study (Finucane et al., 2018) were also included. Tissues in bold black are those with higher enrichment than corresponding anti-target cell type at nominal significance. Tissues in bold red are those with higher enrichment than corresponding anti-target cell type at significance after FDR correction.

File Name: Supplementary Data 4

Description: **A)** PGS_{SCZ} predictions in the SCZ-HC cohort of the present study (N_{SCZ} =1927; N_{HC} = 1561) dividing the target sample in male and female cohorts, using different sets of SNPs based on their association with LNL-ISO (Day et al., 2018). The R^2 proposed by Lee (Lee et al., 2012) was used to estimate the proportion of variance explained by the PGS on the liability scale corrected for ascertainment bias due to oversampling of cases relative to the population prevalence. Prevalence of 1% in SCZ was considered. Alternatively, recent estimates of prevalence in Spanish population were also considered as a sensitivity analysis (males = 0.0079; females = 0.0045; extracted from Orrico Sánchez et al (2020). Number of SNPs used in any case, after clumped, is also displayed (N_{SNP}). CI for the increase in R^2 was estimated through bootstrap resampling (N = 5000). In each subset (SCZ[noLNL], SCZ[LNL], SCZ[CONC] and SCZ[DISC]) all subjects, women (N_{SCZ} =674; N_{HC} = 702) and men (N_{SCZ} =1253; N_{HC} = 859) are used to compare the variance explained by PGS depending on sex. **B)** Differences between the distribution of liability R^2 in males and females across each genomic partition was studied with two-sided t-tests after generation of distributions of R^2 derived from bootstrap resampling (N = 5000). P-values and confident intervals (95%) for each distribution are reported. R^2-liabilitues are expressed in percentage (%).

File Name: Supplementary Data 5

Description: Genetic covariance between SCZ and other relevant phenotypes across LNL-ISO annotations. Partial covariances were calculated with GNOVA within SNP subsets from SCZ[ALL], SCZ[noLNL], SCZ[CONC] and SCZ[DISC]. Error bars represent confidence intervals at 95%. Summary data was downloaded from available repositories (supplementary methods 5). Traits and disorders are abbreviated as follows: major depression (MDD), attention and deficit hyperactivity disorder (ADHD), autism spectrum disorders (ASD), Anxiety disorder (ANX)), Bipolar disorder (BP), obsessive compulsive disorder (OCD), alcohol dependence disorder (ALC-DEP), cross-disorder phenotype (CROSS-DIS), neuroticism (NEUR), depressive symptoms (DS), subjective well-being (SWB), psychotic experiences in the general population (PSY-EXP), educational attainment (EA) and body-mass index (BMI).

File Name: Supplementary Data 6

Description: GWAS summary statistics used in this study.

File Name: Supplementary Data 7

Description: Complete results of the MR analyses and further sensitivity analyses. LNL-ISO: Loneliness MTAG GWAS; LNL UKBB: Loneliness UK Biobank GWAS; FF VISITIS: Frequency and Family visits; N HOUSEHOLD : Number of people in household; ABLE: Able to confide in someone close to you.
