# Supplementary material for: Polygenic contribution to the relationship of loneliness and social isolation with schizophrenia
Source: Nat Commun. 2022 Jan 10;13:51. doi: 10.1038/s41467-021-27598-6 (PMC8748758; doi:10.1038/s41467-021-27598-6)
Supplement: Supplementary file 11 — Reporting Summary [file 41467_2021_27598_MOESM11_ESM.pdf]

nature portfolio

Corresponding author(s): Javier Gonzalez-Peñas

Last updated by author(s): Oct 15, 2021

Reporting Summary

Nature Portfolio wishes to improve the reproducibility of the work that we publish. This form provides structure for consistency and transparency in reporting. For further information on Nature Portfolio policies, see our Editorial Policies and the Editorial Policy Checklist.

Statistics

For all statistical analyses, confirm that the following items are present in the figure legend, table legend, main text, or Methods section.

n/a	Confirmed	
☐	☒	The exact sample size (*n*) for each experimental group/condition, given as a discrete number and unit of measurement
☐	☒	A statement on whether measurements were taken from distinct samples or whether the same sample was measured repeatedly
☐	☒	The statistical test(s) used AND whether they are one- or two-sided<br>*Only common tests should be described solely by name; describe more complex techniques in the Methods section.*
☐	☒	A description of all covariates tested
☐	☒	A description of any assumptions or corrections, such as tests of normality and adjustment for multiple comparisons
☐	☒	A full description of the statistical parameters including central tendency (e.g. means) or other basic estimates (e.g. regression coefficient) AND variation (e.g. standard deviation) or associated estimates of uncertainty (e.g. confidence intervals)
☐	☒	For null hypothesis testing, the test statistic (e.g. *F*, *t*, *r*) with confidence intervals, effect sizes, degrees of freedom and *P* value noted<br>*Give P values as exact values whenever suitable.*
☒	☐	For Bayesian analysis, information on the choice of priors and Markov chain Monte Carlo settings
☐	☒	For hierarchical and complex designs, identification of the appropriate level for tests and full reporting of outcomes
☐	☒	Estimates of effect sizes (e.g. Cohen's *d*, Pearson's *r*), indicating how they were calculated

Our web collection on statistics for biologists contains articles on many of the points above.

Software and code

Policy information about availability of computer code

Data collection	No software has been used for data collection.
Data analysis	R 4.0.2 was used for all statistical analyses<br>SNP were pruned using PLINK v.1.9 with r2 < 0.1 in 500 SNP (--indep-pairwise 500 1 0.1). We used the 10 first MDS components as covariates for polygenic score prediction in the case - control cohort<br>To obtain SNP heritability estimates related to annotations studied here, we followed a recommended procedure (https://github.com/bulik/ldsc/wiki/Partitioned-Heritability).<br>We performed bed file intersection with Bedtools, using the --intersectBed command.<br>We ran LDSC using associated data files from phase 3 of the 1000 Genomes Project.<br>We studied partial correlations using GNOVA (https://github.com/xtonyjiang/GNOVA).<br>We used Mendelian Randomization to investigate the bidirectional causal relationships. Five MR methods (Inverse Variance Weighted (IVW), Weighted Median, MR-Eggers, Simple Mode and Weighted mode) were conducted in the R package TwoSampleMR v.0.5.3 (https://github.com/mrcieu/TwoSampleMR) with the default settings. Additionally, we conducted Mendelian Randomization Pleiotropy RESidual Sum and Outlier (MR-PRESSO)(https://github.com/rondolab/MR-PRESSO) and a MR latent-model method (CAUSE) (https://jean997.github.io/cause/).

For manuscripts utilizing custom algorithms or software that are central to the research but not yet described in published literature, software must be made available to editors and reviewers. We strongly encourage code deposition in a community repository (e.g. GitHub). See the Nature Portfolio guidelines for submitting code & software for further information.

Data

Policy information about availability of data

All manuscripts must include a data availability statement. This statement should provide the following information, where applicable:

- Accession codes, unique identifiers, or web links for publicly available datasets
- A description of any restrictions on data availability
- For clinical datasets or third party data, please ensure that the statement adheres to our policy

COGER ESTO DE LO QUE HE CORREGIDO EN EL MANUSCRITO

Field-specific reporting

Please select the one below that is the best fit for your research. If you are not sure, read the appropriate sections before making your selection.

☒ Life sciences ☐ Behavioural & social sciences ☐ Ecological, evolutionary & environmental sciences

For a reference copy of the document with all sections, see nature.com/documents/nr-reporting-summary-flat.pdf

Life sciences study design

All studies must disclose on these points even when the disclosure is negative.

Sample size	The loneliness-isolation composite phenotype of the UK Biobank study had an effective sample size of 487,647 individuals (Day et, al. 2018). For the remaining phenotypes we used all data available at the UK Biobank. We did not perform sample-size calculations, but this phenotype showed an adequate power for identifying loci with small effects at a genome-wide significant threshold of 5x10-8. For the independent cohort we used CIBERSAM case-control sample of 1927 schizophrenia cases and 1561 healthy controls.
Data exclusions	Information about the samples used in the study can be found in Supplementary Data 6.<br><br>We used the Loneliness and Isolation MTAG GWAS summary statistics from the study by Day et al. 2018, which did not exclude participants with mental disorders. The authors performed a sensitivity analysis excluding participants with self-reported depression, with no appreciable change in test statistics across any of the identified 15 loci, thus supporting that these loci do not influence loneliness through depression.<br><br>UK Biobank participants have European ancestry. For the rest of phenotypes, we used European based GWAS data, except in the case of schizophrenia, since it is the most frequently used phenotype with > 96% European population (Ripke et, al. 2014).
Replication	No replication was done. Polygenic score predictions were performed in an independent cohort.
Randomization	n/a: We did not use an experimental design
Blinding	n/a: We did not use an experimental design

Reporting for specific materials, systems and methods

We require information from authors about some types of materials, experimental systems and methods used in many studies. Here, indicate whether each material, system or method listed is relevant to your study. If you are not sure if a list item applies to your research, read the appropriate section before selecting a response.

Materials & experimental systems

n/a	Involved in the study	
☒	☐	Antibodies
☒	☐	Eukaryotic cell lines
☒	☐	Palaeontology and archaeology
☒	☐	Animals and other organisms
☐	☒	Human research participants
☒	☐	Clinical data
☒	☐	Dual use research of concern

Methods

n/a	Involved in the study	
☒	☐	ChIP-seq
☒	☐	Flow cytometry
☒	☐	MRI-based neuroimaging

Human research participants

Policy information about studies involving human research participants

Population characteristics	The CIBERSAM consortium (Spain, https://www.cibersam.es/en) collected blood samples of 2,145 individuals with a DSM-IV-TR diagnosis of schizophrenia spectrum disorders. After QC filtering, genetic data of 1927 cases (65% males) and 1561 healthy controls (55% males), with a mean age of 33.15, were used for subsequent analysis as an independent case-control sample.<br><br>The description of the remaining cohorts used in the study is available in Supplementary Data 6.
Recruitment	UK Biobank is a well-described cohort. Participants were recruited between 2006 and 2010. See further details on UK biobank recruitment on their website: https://www.ukbiobank.ac.uk/<br>Recruitment of the CIBERSAM consortium sample was performed in 8 different Spanish hospitals, details can be found at: (https://www.cibersam.es/en)<br>Description of the rest of the cohorts is available in Supplementary table 6
Ethics oversight	The UK Biobank study was approved by the North West Multicentre Research Ethics Service.<br>The CIBERSAM study has received approval from the following ethical committees: Comité de Ética de la Investigación con Medicamentos del Hospital Gregorio Marañón, Comité Autonómico de Ética de la Investigación de Galicia, Comisión de Bioética de la Universidad de Barcelona (CBUB), Comité Ético de Investigación-OSI ARABA, Comité de Etica de la Investigación del Principado de Asturias, Comité de Etica de la Investigación con Medicamentos de Cantabria, Comité Ético de Investigación con Medicamentos del Hospital Clínico Universitario de Valencia, and Comitè d'Ètica d'Investigació Clínica de l'Hospital Universitari de Sant Joan de Reus.

Note that full information on the approval of the study protocol must also be provided in the manuscript.
